# Risk Factors, Clinical Presentation, and Outcome of *Acinetobacter baumannii* Bacteremia

**DOI:** 10.3389/fcimb.2017.00156

**Published:** 2017-05-04

**Authors:** Tala Ballouz, Jad Aridi, Claude Afif, Jihad Irani, Chantal Lakis, Rakan Nasreddine, Eid Azar

**Affiliations:** ^1^Department of Infectious Diseases, Saint George Hospital University Medical Center and University of BalamandBeirut, Lebanon; ^2^Department of Family Medicine, Saint George Hospital University Medical Center and University of BalamandBeirut, Lebanon; ^3^Faculty of Medicine, University of BalamandBeirut, Lebanon

**Keywords:** *Acinetobacter baumannii*, extensive drug resistance, bacteremia, sepsis, risk factors, outcome

## Abstract

Infections caused by *Acinetobacter baumannii* (AB), an increasingly prevalent nosocomial pathogen, have been associated with high morbidity and mortality. We conducted this study to analyze the clinical features, outcomes, and factors influencing the survival of patients with AB bacteremia. We retrospectively examined the medical records of all patients developing AB bacteremia during their hospital stay at a tertiary care hospital in Beirut between 2010 and 2015. Ninety episodes of AB bacteremia were documented in eighty-five patients. Univariate analysis showed that prior exposure to high dose steroids, diabetes mellitus, mechanical ventilation, prior use of colistin and tigecycline, presence of septic shock, and critical care unit stay were associated with a poor outcome. High dose steroids and presence of septic shock were significant on multivariate analysis. Crude mortality rate was 63.5%. 70.3% of the deaths were attributed to the bacteremia. On acquisition, 39 patients had septicemia. Despite high index of suspicion and initiation of colistin and/or tigecycline in 18/39 patients, a grim outcome could not be averted and 37 patients died within 2.16 days. Seven patients had transient benign bacteremia; three of which were treated with removal of the line. The remaining four did not receive any antibiotics due to withdrawal of care and died within 26.25 days of acquiring the bacteremia, with no signs of persistent infection on follow up. A prolonged hospital stay is frequently associated with loss of functionality, and steroid and antibiotic exposure. These factors seem to impact the mortality of AB bacteremia, a disease with high mortality rate and limited therapeutic options.

## Introduction

*Acinetobacter baumannii* (AB) is an aerobic non-fermenting gram-negative Coccobacillus, emerging as a prominent nosocomial pathogen with enhanced environmental resilience and propensity to develop resistance to commonly prescribed antimicrobials. Infections caused by AB include blood stream infections, ventilator associated pneumonias, urinary tract infections, meningitis, and wound infections (Munoz-Price and Weinstein, 2008). These infections are associated with high morbidity and mortality and contribute to a prolonged hospital stay and high hospital costs (Lee et al., 2007; Sunenshine et al., 2007; Jang et al., 2009; Asim et al., 2016).

Of particular importance is the ability of Acinetobacter to cause blood stream infections, especially in critically ill patients, the clinical course of which may range from a benign transient bacteremia to fulminant septic shock (Seifert et al., 1995).

Previous studies have demonstrated that crude mortality rates in patients with AB bacteremia varied between 30 and 76%, and factors associated with worse prognosis include immunosuppression (Gulen et al., 2015; Townsend et al., 2015; Gu et al., 2016), drug resistance (Lee et al., 2007; Sunenshine et al., 2007; Fu et al., 2015; Guo et al., 2016), severity of underlying illness (Seifert et al., 1995; Chopra et al., 2013, 2014; Nutman et al., 2014), inappropriate antimicrobial therapy (Esterly et al., 2011; Huang et al., 2012; Shorr et al., 2014; Freire et al., 2016), septicemia (Huang et al., 2012; Ñamendys-Silva et al., 2015; Freire et al., 2016), and prior antibiotic exposure (Chopra et al., 2013, 2014; Gu et al., 2016; Liu et al., 2016).

**Figure 1:**
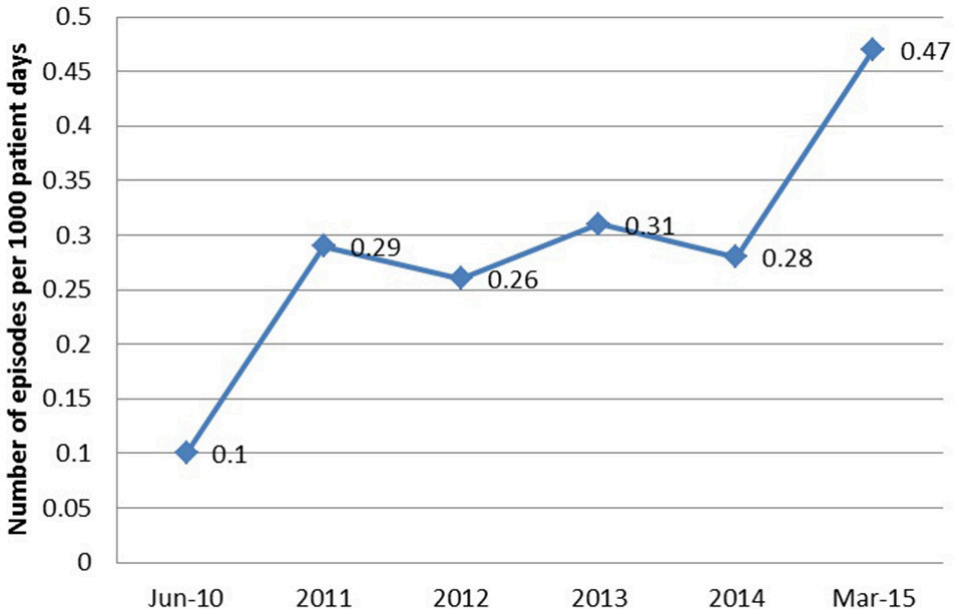
**The annual incidence of *Acinetobacter baumannii* bacteremia from June 2010 to March 2015**.

The epidemic of AB at our institution began in 2010 as an outbreak in the intensive care unit (ICU), rapidly evolving into an endemic with high level resistance, most likely secondary to selective pressure caused by heavy carbapanem usage. Despite implementation of infection control practices and antibiotic restriction, the incidence of AB bacteremias continued to increase (Figure 1). So we conducted this study to analyze the clinical outcomes and risk factors predicting mortality in patients with *Acinetobacter bacteremia*.

## Materials and methods

### Study design

For this purpose, we designed and conducted a retrospective observational study at Saint George Hospital University Medical Center (SGHUMC), a 400 bed tertiary medical center in Beirut, Lebanon. We reviewed the medical records of patients who were admitted between June 2010 and March 2015 and developed *Acinetobacter baumannii* (AB) bacteremia.

### Data collection

An extensive data collection sheet was designed and looked at demographic factors such as age, gender, dates of admission and discharge, admission diagnosis, functional status at admission, and at acquisition of AB. We also gathered data on medical comorbidities, critical care unit stay, invasive procedures prior to acquisition (PTA) of bacteremia, antibiotic and steroid exposure, prior AB infection, sources and clinical manifestation of bacteremia, antimicrobial susceptibility, treatment (time of initiation, doses, routes), and outcome.

### Organism identification and resistance profile classification

At patient bedside, the blood samples were collected directly into BACTEC® culture vials (Becton Dickinson, Heidelberg, Germany). Antimicrobial susceptibility was performed by disc diffusion test and results were interpreted according to Clinical Laboratory Standards Institute Criteria. Extensive drug resistance (XDR) was defined as non-susceptibility to at least one agent in all but two or fewer antimicrobial classes.

### Definitions

Functionality of the patients was obtained according to the Eastern Cooperative Oncology Group (ECOG) performance status: (0) Fully active, able to carry on all pre-disease performance without restriction, (1) Restricted in physically strenuous activity but ambulatory and able to carry out work of a light or sedentary nature, e.g., light house work, office work, (2) Ambulatory and capable of all self-care but unable to carry out any work activities; up and about more than 50% of waking hours (3) Capable of only limited self-care; confined to bed or chair more than 50% of waking hours (4) Completely disabled; cannot carry on any self-care; totally confined to bed or chair, (5) Dead.

High dose steroid was noted to be any dose of steroid of ≥1 mg/kg/day equivalent of prednisone. Previous antibiotic therapy was defined as receiving any systemic antibiotic prior to the positive blood culture for more than 48 h. A critical care stay was noted when a patient had a stay at the Intensive Care Unit (ICU), the Cardiac Care Unit (CCU), or the Cardiothoracic Surgery Unit (CSU) for more than 24 h. Neutropenia was defined as an absolute neutrophil count <1,500 cells/mm^3^. Antimicrobial treatment was defined as “active” if the antibiotics which were administered within 24 h of bacteremia onset, included at least one antibiotic that was active *in vitro*. Bacteremias were classified as primary and secondary. A bacteremia was considered to be secondary when the source is known (pulmonary, gastrointestinal, urinary, wound). A primary bacteremia was either line related or when no apparent source was evident and it was assumed that the patient acquired AB bloodstream infection through colonization of their skin. Septic shock was defined as persistent hypotension despite fluid replacement, requiring vasopressors. We chose in-hospital mortality as the primary outcome measure. A panel of two infectious diseases physicians, two clinical fellows, and a research fellow reviewed the patients' medical records and causes of death were divided into unrelated and related. Unrelated was defined as death that was completely separate from the bacteremia event, related death was defined as death directly due to the AB bacteremia event.

### Statistical analyses

Continuous variables were presented by mean values ± standard deviation and interquartile ranges while categorical ones were presented by percentages. Categorical variables were analyzed using Pearson's chi-square test and continuous variables were analyzed using Student's *t*-test or Mann–Whitney test. To determine independent risk factors for mortality, a multiple logistic regression analysis was used to control for the effect of confounding variables. The results of this regression analyses were reported as adjusted odds ratio (OR) with 95% confidence interval (CI). Time to mortality defined as length of stay post acquisition of bacteremia, in related and unrelated death was analyzed using Kaplan Meier survival analysis, and the log rank test was used for comparison between the two groups. A *p* < 0.05 was considered statistically significant. All statistical analyses were performed using SPSS software version 20.0.

## Results

**Figure 2:**
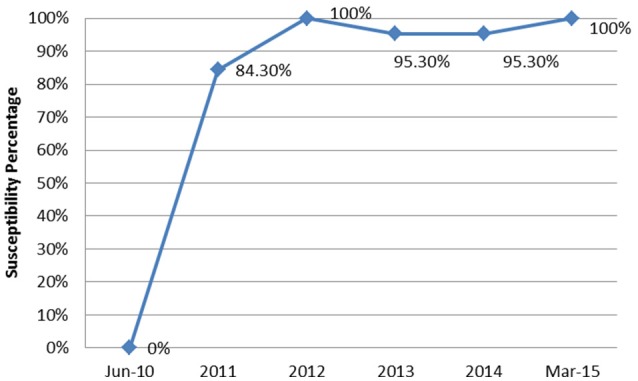
**The annual trend of the extensively drug resistant rate of *Acinetobacter baumannii* isolates from June 2010 to March 2015**.

From June 2010 to March 2015, a total of 90 episodes of AB bacteremias were documented with an increasing incidence from 0.1 per 1,000 patient days in 2010 to 0.47 in 2015 (Figure 1). This was associated with an increase in the resistance profile of AB, where in 2010 all isolates were susceptible, but by 2015 all isolates became XDR (Figure 2).

### Antimicrobial susceptibility

We found that the majority of our isolates were carbapenem resistant (82/90). Seventy-nine isolates (87.7%) were deemed to be XDR, as defined in the Methods Section. Tigecycline had the highest level of *in-vitro* sensitivity at 83.3%. The isolates were highly resistant to piperacillin/tazobactam (87/90), ciprofloxacin (86/90), ceftazidime (85/90), cefepime (83/90), trimethoprim/sulfamethoxazole (82/90), and amikacin (81/90). All isolates were sensitive to colistin by disc diffusion. Moreover, colistin susceptibility by broth microdilution was done in five isolates and all were found to be susceptible.

### Patient characteristics

Demographics and baseline characteristics of the study population are shown in Table 1. Mean age of the patients was 67.14 ± 20.34 years (range 8 days–92 years). The bacteremia was mostly prevalent (54.5%) in the 70–90 years age group. 62.3% of the study population was male.

**Table 1:** **Demographics, clinical characteristics, and exposure prior to *Acinetobacter baumannii* bacteremia event^a^**.

**Variables**	**Number**
Age, mean (SD)	67.14 ±20.34
**Sex (%)**	
Male	53 (62.3)
Female	32 (37.6)
**Comorbidities (%)**	
Hypertension	57 (67)
Malignancies	38 (44.6)
Hematologic	19 (22.3)
Acute leukemia	8 (9.5)
Chronic lymphocytic leukemia	2 (2.3)
Lymphoma	5 (5.9)
Myelodysplastic syndrome	2 (2.3)
Multiple myeloma	2 (2.3)
Solid	19 (22.3)
Diabetes mellitus	25 (29.4)
Coronary artery disease	25 (29.4)
Congestive heart failure	10 (11.7)
Renal disease	15 (17.6)
Dyslipidemia	11 (12.9)
Autoimmune diseases	6 (7)
Lung disease	18 (21.2)
Cerebral vascular accident	6 (7)
Neutropenia prior to event^b^ (%)	13 (15.3)
**ECOG (on admission) (%)**	
1	7 (8.2)
2	14 (16.5)
3	24 (28.2)
4	37 (43.6)
N/A	3 (3.5)
**ECOG (on acquisition) (%)**	
1	3 (3.5)
2	5 (5.9)
3	10 (11.8)
4	64 (75.3)
N/A	3 (3.5)
**Invasive procedures prior to event^b^ (%)**	
Foley	68 (76.4)
Central lines	68 (76.4)
Nasogastric tube	40 (44.9)
Mechanical ventilation	40 (44.9)
Drains	19 (21.1)
Tracheostomy	7 (7.9)
TPN (%)	16 (17.8)
**Antibiotic exposure (%)**	
Prior to admission	25 (27.8)
Prior to event^b^	84 (93.3)
High dose steroid exposure prior to event^b^	53 (58.9)
CC unit stay prior to event^b^	60 (66.7)
LOS prior to event, mean, days^b^	18.97 ±16.79

*SD, standard deviation; ECOG, Eastern Cooperative Oncology Group; TPN, total parenteral nutrition; CC, critical care; LOS, length of stay*.

a *Data are presented as n (%), unless otherwise specified*.

b *Variables are presented by episodes (n = 90), otherwise they are presented by patients (n = 85)*.

On admission, 24 patients (28.2%) had an ECOG of 3, being confined to bed or chair for more than half of the day. Thirty-seven patients (43.5%) had an ECOG of 4, with inability to carry any daily life activities. On acquisition of the bacteremia, 75% of the patients had an ECOG of 4.

Seventy-one patients (83.5%) had at least one comorbidity, hypertension being the most prevalent (67%). Thirty-eight patients (44.6%) had a malignancy, half of which were hematological.

Patients spent a mean duration of 18.97± 16.79 days in the hospital before developing AB bacteremia.

Seventy-seven (90.5%) patients had at least one invasive procedure PTA, most commonly foley and central catheters.

67.7% of the episodes in 58 patients were preceded by a critical care (CC) unit stay. Mean time interval from CC unit admission to developing bacteremia was 9.94 ± 8.32 days.

Twenty-five patients had antibiotic exposure prior to their admission to the hospital and 93.3% of the events were preceded by exposure to at least one antimicrobial (Table 2). Prior exposure to colistin and/or tigecycline was documented in 25 events; 15 of which were for a prior AB infection. Otherwise, they were given empirically.

**Table 2:** **Antibiotic exposure prior to *Acinetobacter baumannii* bacteremia event (*N* = 90)**.

**Antibiotic**	**Number (%)**
Quinolones	32 (35.5)
Antipseudomonal cephalosporins (cefepime, ceftazidime)	15 (16.7)
Carbapenems (meropenem, imipenem)	59 (65.6)
Colistin	17 (18.8)
Tigecycline	16 (17.8)
Other beta lactams (piperacillin/tazobactam, amoxicillin, ceftriaxone)	31 (34.4)
Aminoglycosides	7 (7.8)
Metronidazole	35 (38.9)
Glycopeptides	38 (42.2)

Fifty patients received high dose steroids, most commonly for sepsis, for a mean duration of 8.4 ± 11.21 days. The indications for steroids are listed in Table 3.

**Table 3:** **Indications for high dose steroid exposure (*N* = 53)**.

**Indication**	**Number (%)**
Sepsis	15 (28.4)
Bronchospasm	8 (15.1)
Chemotherapy	8 (15.1)
ARDS	4 (7.5)
Immune (MAS, SLE)	6 (11.3)
More than one indication	8 (15.1)
Other	4 (7.5)

*ARDS, acute respiratory distress syndrome; MAS, macrophage activating syndrome; SLE, systemic lupus erythematosis*.

### Event characteristics and outcome

Clinical characteristics of bacteremia events are listed in Table 4.

**Table 4:** **Clinical characteristics of the *Acinetobacter baumannii* bacteremia events (*N* = 90)**.

**Variables**	**Number (%)**
**Source**	
Primary	57 (63.3)
Secondary	
Lungs	20 (22.2)
Urine	3 (3.3)
Intraabdominal	5 (5.6)
Wound/Soft tissue	5 (5.6)
**Resistance profile**	
XDR	82 (91.1)
Non-XDR	8 (8.9)
Septic shock on acquisition	41 (45.6)
**Active treatment**	
Within 24 h of positive blood culture results	32 (35.6)

*XDR, extensively drug resistant*.

Fifty-seven (63.3%) bacteremia events were considered primary, and thirty-three (36.7%) were secondary to a source of infections, lungs being the most common. Septic shock was present at the time of acquisition in 41 events (45.5%).

**Figure 3:**
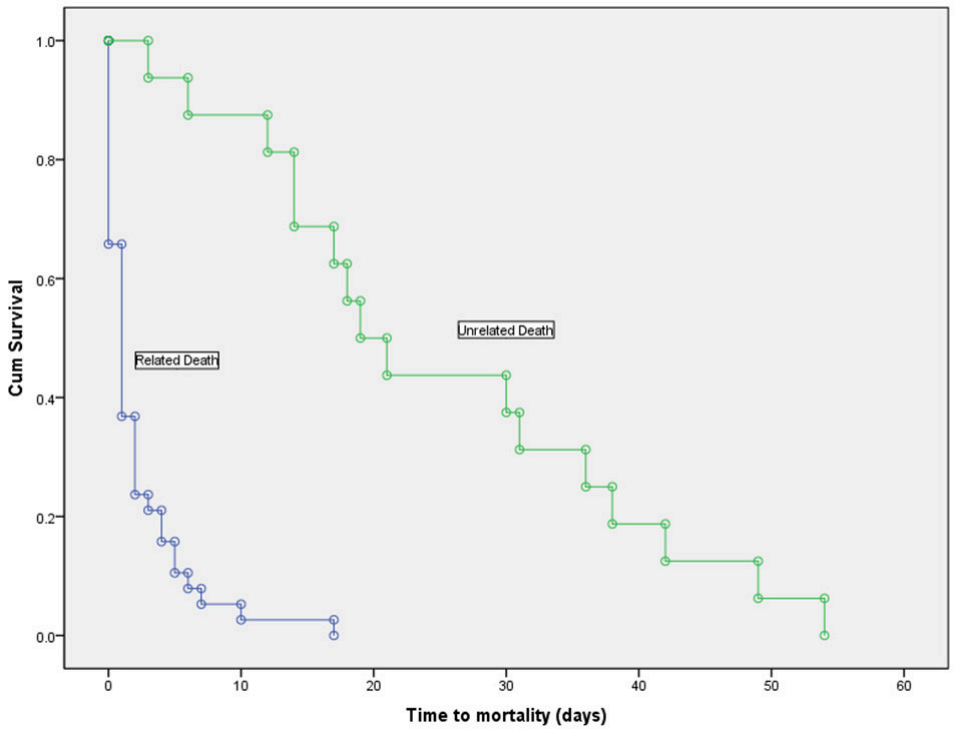
**Kaplan-Meier survival curve in deceased patients with *Acinetobacter baumannii* bacteremia, showing a significantly shorter time to mortality in patients who died due to the bacteremia vs. those who died from unrelated causes (log-rank test, *p* < 0.001)**.

Overall, 54 patients (63.5%) died during their hospitalization with a mean duration of infection to death of 16.03 ±20.31 days. Thirty-eight patients died from causes directly related to the bacteremia, while 16 died from unrelated causes. Figure 3 shows the duration of survival which was significantly shorter in patients who died from related causes (2.16 ± 3.36 vs. 25.25 ± 15.25, *p* < 0.001).

We described three outcomes after acquisition of the bacteremia. The first was where patients recovered without administration of antibiotics (seven patients); three of them were effectively treated by removal of the offending line. The remaining four patients had documented bacteremia and did not receive any treatment; they died after a mean duration of 26.25 days after withdrawal of their care as by the family's wishes. All seven patients had no evidence of persistent signs of infection as by their daily follow up. The second group included 39 patients, with fulminant sepsis on acquisition. Thirty-seven or Thirty-nine died and the time to death was as short as 2.1 days even in those who received active treatment (17/37). The remaining 41 patients recovered from the bacteremia after receiving appropriate antibiotics. Of those, 13 patients died from unrelated causes within a mean of 24.9 days from the onset of bacteremia.

Five patients had two episodes of bacteremia during their hospital stay; of those three died, two of which were from causes related to the bacteremia.

Active treatment description is shown in Table 5. Active antibiotics were administered in 32 episodes in thirty patients within 24 h of positive culture results. Of those patients 24 died. Eight patients died prior to initiation of appropriate treatment.

**Table 5:** **Active antibiotics used in the treatment of *Acinetobacter baumannii* bacteremia**.

**Treatment**	**Number (*n* = 32)**
Colistin	18
Tigecycline	9
Colistin and Tigecycline	5

### Risk factors for mortality

Characteristics of patients stratified by mortality are shown in Table 6.

**Table 6:** **Demographic and clinical characteristics of patients with *Acinetobacter baumannii* bacteremia stratified by all cause in hospital mortality (*N* = 90)**.

**Characteristics**	**Survivors**	**Non-survivors**	***p-values***
**Demographics**			
Age, mean, year (SD)	67.33 ±13.99	71.85 ±16.77	
Male sex (%)	22	33	NS
LOS prior to event, median, days (SD)	12.3 ±11.65	22.41 ±18.05	0.002
**Comorbidities (%)**			
Hypertension	22	39	NS
Malignancies			
Hematologic	6	13	NS
Solid	10	11	NS
Diabetes mellitus	5	21	0.044
Coronary artery disease	11	14	NS
Congestive heart failure	4	6	NS
Renal disease	2	13	NS
Dyslipidemia	4	7	NS
Autoimmune diseases	2	4	NS
Lung disease	7	11	NS
Cerebral vascular accident	2	4	NS
Neutropenia	3	10	NS
High dose steroids	9	44	<0.001
**Invasive procedures prior to event (%)**			
Foley	21	47	NS
Central lines	19	49	NS
Nasogastric tube	11	29	NS
Mechanical ventilation	8	32	0.005
Drains	7	12	NS
Tracheostomy	1	6	NS
Total parenteral nutrition	5	11	NS
CC unit stay	16	44	0.03
XDR profile	28	54	NS
Infection source			
Primary	22	35	NS
Secondary	10	23	
Prior exposure to colistin and/or tigecycline	4	21	0.019
Septic shock at time of acquisition	2	39	<0.001

*SD, standard deviation; LOS, length of stay; CC, critical care*.

No significant difference in demographic characteristics, XDR profile and sources of bacteremia between survivors and non-survivors was found. Patients who did not survive had a longer pre-acquisition median hospital LOS than patients who survived (22.41 vs. 12.3 days, *p* = 0.002). Deceased patients were also more likely to have diabetes mellitus, prior exposure to high dose steroids, received colistin and/or tigecycline PTA, have septic shock at time of bacteremia, and to have stayed longer in a CC unit, with a higher rate of mechanical ventilator use.

In the multivariate analysis, the risk factors independently associated with mortality in patients with AB bacteremia were high dose steroids and septic shock (Table 7).

**Table 7:** **Logistic regression analysis of predictors on all-cause in-hospital mortality among patients with *Acinetobacter baumannii* bacteremia**.

**Predictors**	**Univariate analysis**		**Multivariate analysis**	
	**OR (95%CI)**	***P***	**OR (95%CI)**	***p***
Septic shock	44.2 (9.42–207.3)	<0.001	90.91(11.45–719.12)	<0.001
High dose steroids	6.31 (2.48–16.02)	<0.001	7.74 (1.634–36.6)	0.01
Prior exposure to colistin &/or tigecycline	3.94 (1.19–12.99)	0.019		
CC unit stay	3.13 (1.25–7.83)	0.03		
Mechanical ventilation	3.89 (1.54–9.99)	0.004		
Diabetes mellitus	2.94 (1.04–8.29)	0.041		

*OR, odds ratio; CI, confidence interval; CC, critical care*.

The appropriateness of antimicrobial therapy was not associated with a better outcome since 80% of the patients who had received appropriate antimicrobial therapy within 24 h of positive culture results died compared to 46.8% of those who did not (*p* = 0.003).

## Discussion

In our study, we aimed to identify the clinical characteristics and prognostic factors in patients with AB bacteremia.

In concordance with previous reports (Esterly et al., 2011; Chopra et al., 2013, 2014; Nutman et al., 2014; Fu et al., 2015; Gulen et al., 2015; Gu et al., 2016; Guo et al., 2016; Liu et al., 2016), we found that patients with compromised baseline mobility who are barely capable of carrying any life activities as by their ECOG, with longer hospital stay especially in the critical care units, with ≥1 mg/kg/day of steroids and antimicrobial exposure are at risk for AB bacteremia with poor outcome.

The crude mortality rate in our series was 63.5%, comparable to the range of 30–76% reported in literature (Gulen et al., 2015; Leão et al., 2016). However, it has been difficult to distinguish between attributable mortality and that attributed to the underlying illnesses and comorbid conditions of patients. In a previous case control study, Jang et al. reported that the underlying illnesses seemed to play a more pivotal role than the infection itself as a cause of death (Jang et al., 2009). Attributable mortality in our study was 70.3%, showing that there is significant impact of the bacteremia on the outcome.

In our study, all but two patients with septic shock on acquisition of the bacteremia died, within a mean of 2.16 days. It is well-known that the presence of severe sepsis or septic shock in bacterial infections is associated with worse outcomes. However, there has been limited data describing the outcomes of sepsis in AB infections. Lahmer et al. reported six cases of severe sepsis caused by AB, with a 100% mortality rate (Lahmer et al., 2014). Similarly, Leão et al. associated AB infections with lower survival rates in septic ICU patients, when compared to other pathogens (Leão et al., 2016). Contrary to that, a recent cohort of septic ICU patients with AB bacteremia had a mortality rate of 49.6% (Shorr et al., 2014).

On the other end, we observed that a subset of patients had recovered from the bacteremia, despite little or no intervention. This has been previously described by Seifert et al. as “benign transient bacteremia” (Seifert et al., 1995). Though all cases of this transient bacteremia in our study were line related and removal of the line may had averted the sepsis cascade, the virulence factors of the bacteria and immune status of the host may have also played a significant role.

Previous studies have highlighted on the importance of a patient's immune status on the outcome, with prolonged LOS and mortality rates, particularly when a hematological malignancy is present (Nazer et al., 2015; Gu et al., 2016). Though we did not find any significant association between malignancies and mortality, exposure to high dose steroids increased the risk of dying by 7.4-folds. Townsend et al. (2015) in a recent publication similarly associated steroids with increased mortality.

Patients with prior exposure to colistin and/or tigecycline had appeared to have a worse outcome. One potential explanation is that previous antibiotic therapy might have an effect on selecting and expressing more virulent bacteria (Gordon and Wareham, 2010). However, this was not shown to be an independent risk factor on multivariate analysis, most likely due to the small sample size.

A rapid rise of resistance was noted over the study period, with an overall XDR rate of AB isolates of 87.7%. Yet, we could not correlate between drug resistance and mortality, which could be due to the small number of patients with non XDR AB. In literature, the effect of antibiotic resistance on survival is still a subject of debate. While some authors have argued against any attributed mortality due to resistance (Gu et al., 2016), others have suggested that resistant AB may lower survival either directly by enhanced virulence, or indirectly by delaying the onset of active antimicrobial treatment (Esterly et al., 2011; Huang et al., 2012; Lee et al., 2012; Liu et al., 2015).

In our cohort, administration of appropriate treatment within 24 h of bacteremia was associated with worse outcome. Even when XDR AB was highly suspected, the initiation of active antibiotics did not affect the outcome as much as the critical septic condition of the patient, due to limited time. On the other hand, a subset of non-septic patients who grew AB in blood cultures taken as part of the workup of fever in a hospitalized patient and did not receive active treatment against XDR AB, remained in relatively good clinical conditions. These two extremes of clinical outcome made the analysis of the effect of appropriate antibiotics complicated and may be the reason for opposing results in literature. Lee et al. postulated that inappropriate antimicrobial therapy may be less detrimental in patients who are not severely ill and in the most severely ill with short life expectancies (Lee et al., 2012). Similar results were reported by Lim et al that suggested that host factors and severity of infections reflected by APACHE are the main determinants of the outcome rather than the use of active therapy (Lim et al., 2011). Contrary to that, others have suggested that inactive microbial therapy in patients with AB bacteremia is associated with mortality, and that early initiation of appropriate antimicrobial therapy would lead to a better outcome (Nutman et al., 2014; Shorr et al., 2014).

The present study has several limitations that must be acknowledged, mainly due to its retrospective nature and small sample size. However, to eliminate potential biases, we gathered all the data needed from computerized records through a standardized data collection sheet and performed multivariate analysis to discover independent risk factors. The presence of malignancies in 44.6% of our study population might be an overrepresentation of our hospitalized patients, in the absence of a control group. Also, as a single center study, there is lack of generalizability and may not reflect on other different institutions.

In conclusion, a prolonged hospital stay is frequently associated with loss of functionality, and steroid and antibiotic exposure. These factors seem to impact the mortality of AB bacteremia, a disease with high mortality rate and limited therapeutic options. We believe that improving outcome seems to lie more in the prevention of acquisition of AB bacteremia rather than in treating the consequences.

## Ethics statement

This study was carried out in accordance with the recommendations of “Good Clinical Practice Guidelines” as defined by the U.S. Food and Drug Administration under the code of Federal Regulations (21CFR Parts 50 and 56; 45 CFR Part 46) and International Conference on Harmonization (ICH) Guidelines (Section E6) and applicable local laws and regulations. The Institutional Review Board at University of Balamand-Faculty of Medicine and Medical Sciences approved this study protocol and granted a waiver of informed consent due to the retrospective nature of the study; where data collection occurred after the patients left the hospital or died. Patient medical records and information were anonymized and de-identified prior to analysis.

## Author contributions

EA and CA designed the study; JA gathered the data, JI, CL, and TB analyzed the data; TB wrote the main manuscript text and prepared figures. All authors reviewed the manuscript.

### Conflict of interest statement

The authors declare that the research was conducted in the absence of any commercial or financial relationships that could be construed as a potential conflict of interest.
